# Phosphodiesterase-5 inhibitors for erectile function rehabilitation in patients undergoing nerve sparing radical prostatectomy: a scoping review

**DOI:** 10.1590/0100-6991e-20243757-en

**Published:** 2024-11-07

**Authors:** GABRIEL CARVALHO ANDRADE GADELHA, ARLINDO MONTEIRO DE CARVALHO

**Affiliations:** 1 - Universidade Federal da Paraíba, Curso de Medicina - João Pessoa - PB - Brasil; 2 - Universidade Federal da Paraíba, Departamento de Cirurgia - João Pessoa - PB - Brasil

**Keywords:** Prostatectomy, Erectile Dysfunction, Phosphodiesterase 5 Inhibitors, Prostatectomia, Disfunção Erétil, Inibidores da Fosfodiesterase 5

## Abstract

**Introduction:**

The aim of this study was to conduct a scoping review on the efficacy of phosphodiesterase-5 inhibitors (PDE-5Is) in rehabilitating erectile function in patients undergoing cavernous nerve sparing radical prostatectomy (NSRP).

**Methods:**

The databases used were MEDLINE, EMBASE, CENTRAL, LILACS and Web of Science. Systematic reviews with meta-analyses on the subject were included until March 5, 2024, with no language restrictions. Publications that did not address any of the aforementioned relationships were excluded. The data was organized into tables for descriptive analysis. The methodological quality of the included studies was assessed using the ROBIS tool.

**Results:**

Eight studies were selected and all concluded that the use of PDE-5Is is effective in penile rehabilitation. Only one of the reviews found that use for more than six months was superior to short-term use, and another concluded that daily use was superior to on-demand use. In addition, the articles identified more adverse effects in the experimental group compared to the control group, but without compromising therapeutic adherence. Six of the studies were classified as low risk of bias, while the other two had uncertain risk.

**Conclusion:**

PDE-5Is are effective in restoring erectile function in patients undergoing NSRP, especially when used regularly and over the long term, and follow-up is not hampered by adverse effects. However, due to the scarcity of data, new studies should be carried out to determine the best form of use of these drugs.

## RESUMO

**Introdução:**

O objetivo deste estudo foi realizar uma revisão de escopo sobre a eficácia dos inibidores da fosfodiesterase-5 (PDE-5Is) na reabilitação da função erétil em pacientes submetidos à prostatectomia radical preservadora de nervos cavernosos (PRPN).

**Métodos:**

As bases de dados utilizadas foram MEDLINE, EMBASE, CENTRAL, LILACS e Web of Science. Foram incluídas revisões sistemáticas com meta-análise sobre o tema, até 5 de Março de 2024, sem restrição de idioma. Foram excluídas publicações que não abordassem alguma das relações citadas. Os dados foram organizados em tabelas para realização de análise descritiva. A qualidade metodológica dos estudos incluídos foi avaliado por meio da ferramenta ROBIS.

**Resultados:**

Foram selecionados 8 estudos e todos concluíram que o uso dos PDE-5Is é eficaz na reabilitação peniana. Apenas uma das revisões verificou que o uso por mais de seis meses foi superior ao curto prazo e, outra concluiu que o uso de forma diária foi superior em relação ao uso sob demanda. Além disso, os artigos identificaram mais efeitos adversos no grupo experimental em relação ao grupo controle, mas sem comprometer a adesão terapêutica. Seis dos estudos foram classificados como baixo risco de viés, enquanto os outros dois tiveram risco incerto.

**Conclusão:**

Os PDE-5Is são eficazes na recuperação da função erétil em pacientes submetidos à PRPN, sobretudo quando utilizados de forma regular e a longo prazo, cujo seguimento não é prejudicado pelos efeitos adversos. No entanto, devido à escassez de dados, novos estudos devem ser realizados para determinar a melhor forma de utilização dessas medicações.

## INTRODUCTION

Prostate cancer (PCa) is the second most prevalent neoplasm and the fifth leading cause of cancer mortality among men^1^. It is the leading cause of death associated with malignant tumors in Western countries and mainly affects men between the fourth and sixth decades of life^2^. Screening occurs on an outpatient basis, through digital rectal examination and PSA measurement, and the diagnosis is confirmed by biopsy. The treatment of prostate cancer depends on variables involving the patient and the tumor, such as serum PSA levels, Gleason score, TNM staging, urinary function, comorbidities, and age^1^ ^,^ ^2^.

Radical prostatectomy is a surgical procedure in which the prostate is removed through small incisions in the abdomen or perineum, can be performed openly, laparoscopically, or robotically, and is recommended for patients under 70 years of age, with little or no comorbidity, life expectancy of more than 10 years, and tumors confined to the prostate^2^ ^-^ ^5^. However, these procedures are not free from postoperative complications, such as erectile dysfunction, whose mechanism may be associated with the involvement of the cavernous nerves and involvement of arterial branches of the anterior prostate capsule, which communicate with the cavernous arterial plexus^6^.

The main treatment of erectile dysfunction is through phosphodiesterase 5 inhibitors (PDE-5Is) drugs such as sildenafil, tadalafil, vardenafil, and avanafil, which act to increase arterial vasodilation in the corpora cavernosa and lead to erection^7^. However, neurovascular impairment in the surgical procedure may reduce the responsiveness of one of these drugs, which would reduce its applicability. Among the ways to assess erectile function, there are the International Index of Erectile Function (IIEF) score and positivity in the questions of erection sufficient for vaginal penetration (Sexual Encounter Profile Question 2 - SEP2), long-lasting erection for the sexual course (Sexual Encounter Profile Question 3 - SEP3), and improvement of erectile function with treatment (Global Assessment Question - GAQ)^8^.

Surgical techniques have been improved to reduce postoperative complications, such as nerve-sparing techniques, which are associated with the preservation of the cavernous nerve and, consequently, with the maintenance of erectile function^9^ ^,^ ^10^. Nerve-sparing radical prostatectomy (NSRP) can have different classifications and techniques according to the surgeon, but the main forms of classification take into account the anatomy of dissection, which can refer to the prostate capsule (intrafascial, interfascial, or extrafascial) and the laterality of the preserved nerve, which can be unilateral (UNSRP) and bilateral (BNSRP)^9^ ^,^ ^10^. 

However, even in a successful procedure, there is no guarantee of function recovery, as it may take two years to return to normality and reach less than 20% without medications use. Due to this, the most up-to-date literature recommends immediate intervention with PDE-5 inhibitor, but there is still no consensus on the form and duration of use of these drugs, nor the adverse effects that this therapy may bring^10^ ^,^ ^11^.

In this context and considering the large number of systematic reviews on the subject, the present study aims to perform a scoping review, following the protocol of the Preferred Reporting Items for Systematic Reviews and Meta-Analysis for Scoping Reviews (PRISMA-ScR), on the use of PDE-5Is in the recovery of erectile function in patients undergoing nerve-sparing radical prostatectomy (NSRP). This review is of paramount importance to evaluate the overall efficacy of the class, duration of use (short and long term), and regimen (regular and on-demand), and the presence of adverse effects associated with such drugs, to establish the best therapeutic strategy in the postoperative period of these patients, which remains undefined in the scientific community.

## METHODOLOGY

### Study design and search strategy

Based on the criteria of the PRISMA-ScR, we conducted a scoping review that included systematic reviews with meta-analysis that evaluated the efficacy of the use of phosphodiesterase 5 inhibitors in patients with erectile dysfunction after cavernous nerve-sparing radical prostatectomy^12^. 

This scoping review has been registered with the Open Science Framework (OSF). It followed the guidelines in chapter 10 of the Joanna Briggs Institute (JBI) evidence synthesis manual^13^.

A systematic electronic search was carried out on March 5, 2024, using different databases, including the Medical Literature Analysis and Retrieval System Online (MEDLINE), Excerpta Medica Database (EMBASE), Latin American and Caribbean Literature on Health Sciences (LILACS), Cochrane Central Register of Controlled Trials (CENTRAL), and Web of Science.

To build the search strategy used in each database, were searched for the descriptors of the Medical Subject Headings (MeSH) - used in the MEDLINE, LILACS, CENTRAL and Web Of Science databases - and EmTree - used in EMBASE -, combined with the Boolean operator ‘AND’ to confer greater sensitivity (Table 1)

**Table 1:** Search strategy according to each database used.

DATABASES	SEARCH STRATEGY	APPLIED FILTER
MEDLINE	(("Prostatectomy"[Mesh]) AND "Erectile Dysfunction"[Mesh]) AND "Phosphodiesterase 5 Inhibitors" [Pharmacological Action]	Meta- Analysis
EMBASE	prostatectomy'/exp AND 'erectile dysfunction'/exp AND 'phosphodiesterase v inhibitor'/exp	('meta analysis'/de OR 'meta analysis topic'/de OR 'network meta analysis'/de)
CENTRAL	[Prostatectomy] explode all trees AND [Erectile dysfunction] explode all trees AND [Phosphodiesterase 5 Inhibitors] explode all trees	Cochrane Reviews
LILACS	(prostatectomy OR retropubic prostatectomy OR suprapubic prostatectomy) AND (erectile dysfunction OR male sexual impotence OR male impotence OR impotence) AND phosphodiesterase 5 inhibitors	-
WEB OF SCIENCE	(((ALL=(Prostatectomy)) AND ALL=(Erectile Dysfunction)) AND ALL=(Phosphodiesterase 5 Inhibitors)) AND ALL=(Meta-Analysis)	-

### Eligibility criteria

We included systematic reviews with meta-analysis of randomized controlled trials, without date or language restrictions, evaluating the efficacy of the isolated use of PDE-5Is for the recovery of erectile function (measured by the IIEF-EF score and/or positivity in SEP2, SEP3, or GAQ questions) in patients undergoing nerve-sparing radical prostatectomy, who did not have previous erectile dysfunction, regardless of age or presence of comorbidities. 

We excluded studies involving other modalities of surgery or whose surgical procedure was not specified as nerve preservative, as well as articles that combined other interventions with PDE-5Is or that did not discuss any of the possible relationships mentioned above.

### Study Selection

Two independent reviewers selected the studies through the analysis of titles and abstracts, with conflict resolution by consensus among them. Next, we conducted the full-text screening of the studies selected in the earlier stage, to meet the established eligibility criteria, based on a consensus between the two main reviewers. We used the Covidence tool to manage the references in the execution of this step^14^.

### Data collection and evaluation

Data collection was conducted by two independent reviewers, with conflict resolution through consensus among the authors.

We arranged the extracted data into tables and pre-made forms. We recorded identification information from each study (authors, year of publication), objectives, study population (number of patients studied and laterality of nerve-sparing), as well as exposure-related variables (name of PDE -5Is used in the experimental group, use of regular or on-demand medication, short-term or long-term use, association with other therapies, intervention in the control group) and the possible outcomes evaluated, which were the recovery of erectile function evaluated through the IIEF-EF score and positivity in SEP2, SEP3, and GAQ, in addition to the presence of possible adverse effects.

### Evaluation of methodological quality and bias

The evaluation of the methodological quality of the studies was conducted using the ROBIS - Risk of Bias in Systematic tool^15^. This is one of the main tools used to assess the risk of bias in systematic reviews with meta-analysis^16^. 

This stage was also conducted by two independent reviewers, and conflicts were resolved by consensus among the authors.

### Data synthesis and statistical analysis

A qualitative (descriptive) analysis of the data was performed through tables for the synthesis of the information presented. Quantitative analysis was not conducted due to the heterogeneity of the studies and the scarcity of data.

## RESULTS

### Study Selection

**Figure 1:**
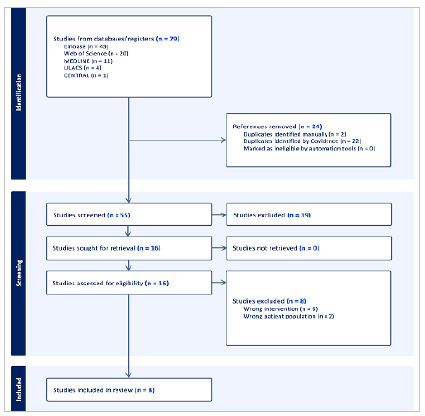
Search strategy results.

The search strategy found 79 studies, of which 24 were duplicates and 39 were excluded during the title and abstract screening, leaving 16 to be fully reviewed. In the end, we excluded eight studies, as they presented deviations in terms of the type of population (not only patients undergoing NSRP) and intervention analyzed (not only the use of PDE-5Is), resulting in eight articles included for review (Figure 1). 

### Characterization and qualitative analysis of studies

The included studies are systematic reviews with meta-analysis of randomized controlled trials (RCTs) in humans. In all articles, the drugs used were sildenafil, tadalafil, and vardenafil, while in six of them avanafil was also evaluated (Cui et al., 2016; Goh et al., 2022; Li et al., 2014; Limocin et al., 2017; Wang et al., 2014; Yang et al., 2021). In addition, the reviews worked with different medication regimes (on-demand, regular, short-term, and long-term). In five of the studies, the controlled group used placebo exclusively (Cui et al., 2016; Goh et al., 2022; Li et al., 2014; Limocin et al., 2017; Tian et al., 2017) and six evaluated possible adverse effects of treatment (Cui et al., 2016; Goh et al., 2022; Li et al., 2014; Qiu et al., 2016; Tian et al., 2017; Wang et al., 2014). We summarized the main information of each article and organized it in a table (Table 2).

**Table 2:** Synthesis of data from each study.

Study	Number of Randomized Controlled Trials	Nerve-sparing laterality (sample)	Intervention	Control	Outcomes measured	Adverse effects
Cui et al (2016)	6	Bilateral (1678)	Sildenafil, tadalafil, vardenafil, avanafil.	Placebo	IIEF-EF, SEP2 and SEP3	Headache, flushing and dyspepsia
Goh et al (2022)	14	Unilateral (678) Bilateral (2144)	Sildenafil, tadalafil, vardenafil, avanafil.	Placebo	IIEF-EF, regular and on-demand use, SEP2 and SEP3	TEAEs, headache, flushing, dyspepsia, and UAS
Li et al (2014)	7	Unspecified (2665)	Sildenafil, tadalafil, vardenafil, avanafil.	Placebo	IIEF-EF, SEP2, SEP3 and GAQ	TEAEs
Limocin et al (2017)	7	Bilateral (2317)	Sildenafil, tadalafil, vardenafil, avanafil.	Placebo	IIEF-EF and SEP3, regular and on-demand use	-
Qiu et al (2016)	14	Unilateral (762) Bilateral (2413)	Sildenafil, tadalafil, vardenafil	Placebo and no treatment	IIEF-EF, regular and on-demand use	TEAEs
Tian et al (2017)	8	Unspecified (1806)	Sildenafil, tadalafil, vardenafil	Placebo	IIEF-EF, regular and on-demand, short- and long-term use	TEAEs
Wang et al (2014)	8	Bilateral (2018)	Sildenafil, tadalafil, vardenafil, avanafil	Placebo and no treatment	IIEF-EF, regular and on-demand use, SEP2, SEP3 and GAQ	TEAEs, headache, flushing, dyspepsia, and UAS
Yang et al (2021)	14	Unilateral (504) Bilateral (2597)	Sildenafil, tadalafil, vardenafil, avanafil	Placebo and no treatment	IIEF-EF, regular and on-demand use	-

Synthesis of data from each study. GAQ: Global Assessment Questionnaire. IIEF-EF: International Index of Erectile Function - Erectile function. SEP2: Sexual Encounter Profile question 2. SEP3: Sexual Encounter Profile question 3. TEAEs: Treatment-emergent adverse events. UAS: Upper airway symptoms.

All studies evaluated the use of PDE-5 inhibitors in relation to the control group in the recovery of erectile function according to the IIEF-EF score. To determine the efficacy of the medications, systematic reviews with meta-analysis used the event rate and the 95% confidence interval, and the inverse of variance (IV) statistical method to evaluate the difference between means and continuous data. Four studies used the random-effects model for analysis (Goh et al., 2022; Qiu et al., 2016; Wang et al., 2014; Yang et al., 2021), while three used the fixed-effects model (Cui et al., 2016; Li et al., 2014; Tian et al., 2017), and Limocin et al. (2017) evaluated the recovery of erectile function as IIEF-EF values equal to or greater than 22 and, therefore, used the Odds Ratio for binary data. 

The articles concluded that PDE-5Is were effective for the rehabilitation of erectile function compared with the control group. Tian et al. (2017) also divided the comparison into short-term (less than or equal to 6 months) and long-term (greater than 6 months) use, both achieving satisfactory results, with higher values for long-term use (Table 3).

**Table 3:** 

Study	PDE-5Is x Control	Regular use vs. Control	On-Demand Use vs. Control	Regular vs. on-demand use
Cui et al (2016)A	4.04 [2.87, 5.22]	-	-	-
Goh et al (2022)A	4.93 [4.14, 5.71]	4.68 [3.89, 5.46]	4.98 [3.57, 6.39]	-
Li et al (2014)A	4.35 [3.42, 5.29]	-	-	-
Limocin et al (2017)B	2.383 [1.924, 2952]	1.512 [1.295, 1.766]	2.663 [2.506, 2.830]	-
Qiu et al (2016) A	4.45 [3.70, 5.19]	4.66 [3.54, 5.79]	4.14 [2.93, 5.36]	3.49 [1.96, 5.02]
Tian et al (2017) < 6 monthsA	2.26 [1.45, 3.08]	4.08 [3.20, 4.97]	2.64 [-0.87, 6.14]	-
Tian et al (2017) > 6 monthsA	4.50 [3.60, 5.40]	4.74 [3.79, 5.69]	-	-0.56 [-9.86, 8.74]
Wang et al (2014) A	5.63 [4.26, 6.99]	4.72 [3.21, 6.23]	5.61 [4.73, 6.50]	-
Yang et al (2021) A	2.67 [1.98, 3.59]	2.12 [1.56, 2.89]	3.00 [1.83, 4.91]	-

A: Difference in the means of the impact on the International Index of Erectile Function - Erectile Function score. B: Odds Ratio for score ≥ 22 of the International Index of Erectile Function - Erectile Function score.

Six studies compared the use of medications on a regular (daily) or on-demand basis with the placebo group, and all results were favorable for the experimental groups (Goh et al., 2022; Limocin et al., 2017; Qiu et al., 2016; Tian et al., 2017; Wang et al., 2014; Yang et al., 2021), with only two articles showing higher values for regular use compared with on-demand use (Qiu et al., 2016; Tian et al., 2017). These two studies also directly compared the two forms of administration and, while Qiu et al. (2016) found better results favoring the regular group, Tian et al. (2017) did not find differences in the IIEF-EF score of each population (Table 3).

Four studies evaluated medications in relation to the control group for positive SEP2 response and five studies for SEP3, and all observed better results in the experimental groups (Cui et al., 2016; Goh et al., 2022; Li et al., 2014; Limocin et al., 2017; Wang et al., 2014) (Table 4). Li et al. (2014) and Wang et al. (2014) also evaluated the positivity in the GAQ response, and both concluded that PDE-5Is were beneficial for this parameter in relation to placebo. Finally, six studies evaluated the presence of adverse effects, such as treatment-emergent adverse events (TEAEs), headache, flushing, dyspepsia, and upper airway symptoms (UAS), which showed higher values in the experimental group compared with the control one (Cui et al., 2016; Goh et al., 2022; Li et al., 2014; Qiu et al., 2016; Tian et al., 2017; Wang et al., 2014) (Table 5).

**Table 4:** 

Study	SEP 2	SEP3	GAQ
Cui et al (2016)A	14.87 [4.57, 48.37]	6.47 [3.00, 13.98]	-
Goh et al (2022)A	2.27 [1.90, 2.86]	2.78 [1.97, 3.91]	-
Li et al (2014)	21.49 [16.36, 26.63]B	17.01 [8.46, 25.56]B	3.50 [2.31, 5.31]C
Limocin et al (2017) RegularA	-	2.024 [1.370, 2.991]	-
Limocin et al (2017) On DemandA	-	2.935 [2.532, 3.403]	-
Wang et al (2014)C	1.63 [1.18, 2.25]	2.00 [1.27, 3.15]	3.53 [2.68, 4.67]

A: Odds Ratio of the response impact on SEP2, SEP3, and GAQ. B: Difference in response impact means in SEP2 and SEP3. C: Relative risk of response impact on SEP2, SEP3, and GAQ. GAQ: Global Assessment Questionnaire. SEP2: Sexual Encounter Profile question 2. SEP3: Sexual Encounter Profile question 3.

**Table 5:** 

Study	TEAEs	Headache	Blush	Dyspepsia	Upper airway symptoms
Cui et al (2015)A	-	2.86 [1.87, 4.39]	5.64 [1.99, 16.01]	4.86 [2.28, 10.36]	-
Goh et al (2022)A	2.91 [1.84, 4.61]	3.38 [2.40, 4.75]	9.44 [4.30, 20.70]	4.49 [2.44, 8.27]	2.59 [1.86, 3.61]
Li et al (2014)B	1.42 [1.21, 1.65]	-	-	-	-
Qiu et al (2016)B	1.68 [1.28, 2.21]	-	-	-	-
Tian et al (2017)A	1.55 [1.26, 1.91]	-	-	-	-
Wang et al (2014)A	2.11 [1.66, 2.67]	2.99 [2.22, 4.04]	4.71 [3.19, 6,95]	3.15 [1.86, 5.35]	2.66 [1.85, 3.84]

A: Odds Ratio of the incidence of adverse effects. B: Relative risk of the incidence of adverse effects. TEAEs: Treatment-emergent adverse events.

### Evaluation of methodological quality, bias and evidence

The evaluation of methodological quality carried with the Risk of Bias in Systematic Reviews (ROBIS) tool found six studies with low risk of bias and two with unclear risk. The reviews were at low risk of bias in data collection, study evaluation, synthesis, and results, but five of the articles were at elevated risk of bias in the identification and selection of studies. Table 6 summarizes the information on the studies’ methodological quality^15^.

**Table 6:** Risk of bias analysis with the ROBIS tool.

	PHASE 2 (RISK OF BIAS)			PHASE 3	
Study	Eligibility criteria for studies	Identification and selection of studies	Data collection and study evaluation	Synthesis and results	Risk of review bias
Cui et al (2016)	B	A	B	B	B
Goh et al (2022)	B	A	B	B	B
Li et al (2014)	B	B	B	B	B
Limocin et al (2017)	B	A	B	B	B
Qiu et al (2016)	?	A	B	B	?
Tian et al (2017)	?	A	B	B	?
Wang et al (2014)	B	B	B	B	B
Yang et al (2021)	B	B	B	B	B

A: High risk of bias. B: Low risk of bias. ?: Uncertain risk of bias.

## DISCUSSION

Phosphodiesterase 5 inhibitor drugs (PDE-5Is) are the first line for the treatment of erectile dysfunction and have their mechanism of action associated with physiological erection, which occurs through nerve impulses to the corpora cavernosa and leads to the production of nitric oxide (NO) by the vascular endothelium. This diffuses through adjacent smooth muscle cells and promotes the activation of the enzyme guanylate cyclase. It converts guanosine triphosphate (GTP) into cyclic guanosine monophosphate (cGMP), which promotes vasodilation and increased arterial flow in the corpora cavernosa, necessary to consolidate the erection itself. Phosphodiesterase 5 (PDE-5), in turn, degrades cGMP into guanosine monophosphate (GMP), which leads to a decrease in vasodilation and so in erection. Thus, these drugs act by inhibiting phosphodiesterase 5, prolonging erection^7^.

In the case of patients with prostate cancer with indication for radical prostatectomy, surgery to preserve the cavernous nerve is increasingly indicated to reduce potential adverse effects, such as urinary incontinence and erectile dysfunction^2^ ^,^ ^9^. However, the combination of both the surgical modality and the postoperative use of PDE-5Is is essential for the maintenance of adequate erectile function^11^.

This is the first scoping review that included systematic reviews with meta-analysis on the subject and found equivalent results in the experimental drug use groups compared with the control groups. All systematic reviews included were in accordance with the current literature and concluded that PDE-5Is are effective for erectile function recovery in all parameters evaluated, whether the IIEF-EF score, or the positivity in the SEP2, SEP3, and GAQ questionnaires, which indicates that the drugs were responsible for ensuring less difficulty for patients to achieve erection, sufficient for vaginal penetration, for a prolonged time for sexual intercourse, and ejaculation, in addition to perceiving the benefit of the treatment for erectile function^8^ ^,^ ^17^ ^-^ ^23^. 

Short- and long-term use were poorly evaluated among the studies, but when analyzed, the use of the drugs for more than six months showed better results, indicating a preference for prolonged treatment^20^.

Regarding regular or on-demand use, for which there is no consensus in the literature, the studies diverged, with most presenting the results of on-demand use in relation to the control higher than the regular use in relation to the control for the IIEF-EF score and for SEP3 positivity, the latter evaluated in a single review^21^. However, when comparing regular use and on-demand use directly, one study obtained better results for regular use, while another did not find differences in the IIEF-EF score^20^ ^-^ ^22^. 

These results imply that the use of PDE-5Is on a regular basis, for a period longer than six months, is the best therapeutic alternative for postoperative erectile recovery, which suggests the need for good patient adherence. However, this comparison was made by few reviews and did not reach significant values in its entirety. Thus, although suggestive, this benefit cannot be confirmed and, therefore, more primary studies and systematic reviews are necessary, with large samples, to define the best way to use these drugs.

On adverse effects, the studies concluded that these were more associated with the use of PDE-5Is, such as Treatment Emergent Adverse Events (TEAEs), which refers to any adverse effect that occurred to patients from their participation in clinical trials. Other symptoms such as headache, flushing, dyspepsia, and symptoms associated with the upper airways, such as rhinosinusitis, were also more associated with the experimental group. However, it is worth noting that all these clinical manifestations did not impair the therapeutic follow-up, that is, regardless of the form of use, whether regular or on-demand, the patients did not abandon treatment, which demonstrates the safety and tolerability of these drugs for postoperative management and ensures good adherence for regular and prolonged use^8^ ^,^ ^23^.

Overall, the systematic reviews evaluated were well executed and followed a safe protocol, with only two of the reviews presenting uncertain risk of bias. However, most studies presented a high risk of bias regarding the identification and selection of studies, mainly due to restrictions imposed (such as language or availability of full text) that were not justified later, and to a lesser extent, due to the low variety of databases and imprecise search strategy^15^.

This study has some limitations, especially related to the included reviews, which presented overlap of primary studies, which gives greater weight to a given clinical trial included in more than one review and may overestimate or underestimate results. The reviews also showed a high risk of study selection bias, and the two articles that compared the regular use of PDE-5Is with the on-demand use were the only ones that showed an unclear risk of bias, due to the eligibility criteria, identification, and selection of the studies, compromising their analysis. Thus, it is not yet possible to establish the best therapeutic strategy for patients undergoing NSRP in a meaningful way. Finally, the heterogeneity of the data made it impossible to conduct a quantitative evaluation, which restricted this study to a description of the main findings.

## CONCLUSION

The maintenance of erectile function after nerve-sparing radical prostatectomy is still a much-reviewed topic. This scoping review points to the effectiveness of phosphodiesterase 5 inhibitors for treating postoperative erectile dysfunction. Prolonged and regular use showed better results when compared with short-term and on-demand use, which implies the need for prolonged, continuous, and well-adhered therapy, which is not impaired by any associated side effects. However, information about the duration and form of use of the drugs is scarce to reach a definitive answer. Thus, randomized clinical trials are necessary, with a larger sample and follow-up time, as well as systematic reviews with meta-analysis of lower risk of bias, especially in the identification and selection of studies, to define the best treatment strategy for these patients.
